# Supplementary material for: Simvastatin therapy attenuates memory deficits that associate with brain monocyte infiltration in chronic hypercholesterolemia
Source: NPJ Aging Mech Dis. 2021 Aug 4;7:19. doi: 10.1038/s41514-021-00071-w (PMC8338939; doi:10.1038/s41514-021-00071-w)
Supplement: Supplementary file 1 — Supplementary Information [file 41514_2021_71_MOESM1_ESM.pdf]

SUPPLEMENTARY MATERIAL

Simvastatin Therapy Attenuates Memory Deficits that Associate with Brain Monocyte Infiltration in Chronic Hypercholesterolemic ApoE$^{-/-}$ Mice

Nicholas Don-Doncow[a,b], Lotte Vanherle[a,b], Frank Matthes[a,b], Sine Kragh Petersen[c], Hana Matuskova[a,b,d,e], Sara Rattik[f], Anetta Härtlova[c] & Anja Meissner[a,b,e]

[a] Department of Experimental Medical Sciences, Lund University, Sweden

[b] Wallenberg Centre for Molecular Medicine, Lund University, Sweden

[c] Department of Microbiology and Immunology, Wallenberg Centre for Molecular and Translational Medicine, University of Gothenburg, Sweden

[d] Department of Neurology, University Hospital Bonn, Germany

[e] German Center for Neurodegenerative Diseases, Bonn, Germany

[f] Department of Clinical Science, Lund University, Sweden

Correspondence to: Anja Meissner; Klinikgatan 32, SE-22184 Lund, Sweden; anja.meissner@med.lu.se; +46 (0)46 22 20641

SUPPLEMENTARY FIGURES

SUPPLEMENTARY FIGURE 1: Cholesterol- and BP-lowering therapy reduce systolic BP in aged ApoE$^{-/-}$ mice. Systolic BP of 12- and 14-months old ApoE$^{-/-}$ mice before and after treatment with BP- and cholesterol-lowering drugs (hydralazine, simvastatin and combination treatment). ApoE – apolipoprotein E, BP_{sys} – systolic blood pressure. *Values expressed in mean ± SEM; N denotes number of independent biological replicates; N=10 for aged control, aged + hydralazine and aged + simvastatin treatment; N=8 for aged + combination treatment; * denotes P ≤ 0.05 relative 12 months (pre-treatment) after Two-way repeated measure ANOVA and Sidak post hoc testing.*

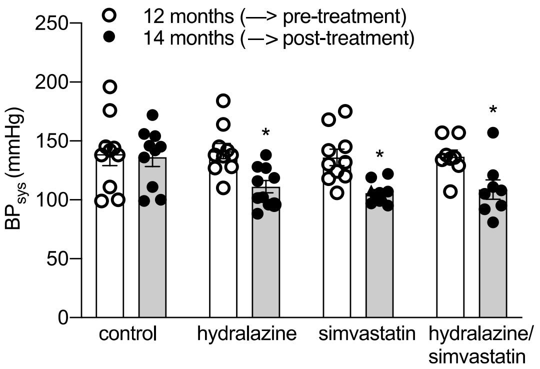

SUPPLEMENTARY FIGURE 2: Region-specific microglia distribution and effect of treatment. **(A)** Total number of CD68+ Iba-1+ cells in the cortex and **(B)** total number of Iba1+ cells in the DG and **(C)** the cortex of 14-months old ApoE$^{-/-}$ mice compared to 4-months old ApoE$^{-/-}$ mice, and the effect of BP- and cholesterol-lowering therapy (hydralazine – green circle, simvastatin – pink circles, combination – orange circles). ApoE – apolipoprotein E, BP – blood pressure, DG – dentate gyrus, Iba-1 – ionized calcium-binding adapter molecule-1. *Values expressed in mean ± SEM; N denotes number of independent biological replicates; N=10 for aged + hydralazine; N=8 for aged control and aged + simvastatin treatment; N=6 for young control and aged + combination treatment; * denotes P ≤ 0.05 relative to young control, & denotes P ≤ 0.05 relative to aged control after Kruskal Wallis followed by Dunn's post hoc testing.*

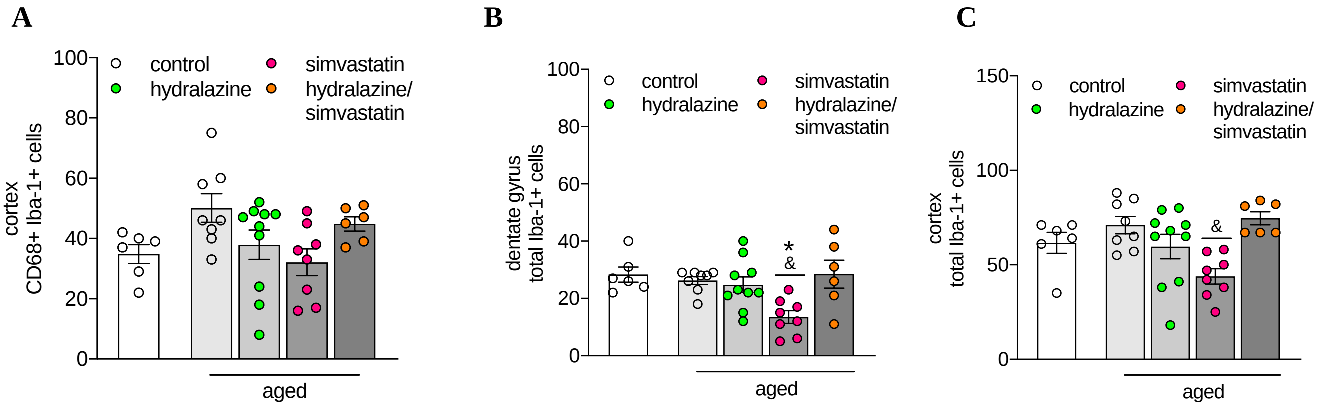

SUPPLEMENTARY FIGURE 3: Simvastatin and Hydralazine treatment lower macrophage activation in primary mouse bone marrow-derived macrophages. Flow cytometry analysis of (**A**) CD86 and (**B**) CD80 responses to LPS, simvastatin and hydralazine in murine bone marrow-derived macrophages, and the effect of BP- and cholesterol-lowering therapy (hydralazine – green circle, simvastatin – pink circles). BP – blood pressure, LPS – lipopolysaccharide. *Values expressed in mean ± SEM; N denotes number of independent biological replicates, N=3 per group; * denotes P ≤ 0.05 relative to untreated control, & denotes P ≤ 0.05 relative to LPS control after one-way ANOVA followed by Tukey's post hoc testing.*

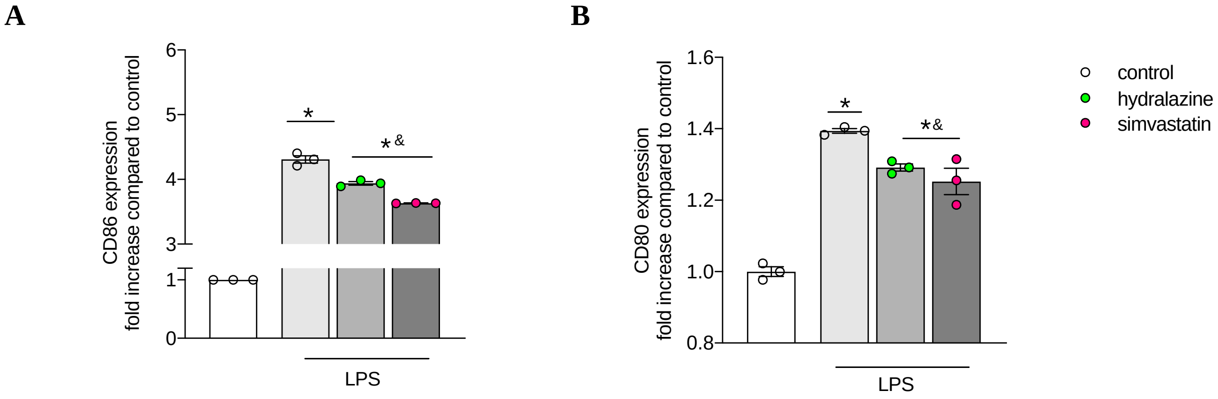

SUPPLEMENTARY FIGURE 4: Hydralazine augments CD3 surface expression in human monocytic THP cells and primary human monocytes as well as monocyte-derived macrophages. Flow cytometric assessment of hydralazine effects on CD3 expression in (**A**) monocytic THP-1 cells, (**B**) PMA-differentiated THP-1 macrophages, (**C**) primary human blood monocytes, and (**D**) primary human monocyte-derived macrophages. **(E)** Representative histograms showing hydralazine responses in THP-1 cells and human primary cells. PMA - phorbol 12-myristate 13-acetate. *Values are expressed in mean ± SEM; N denotes number of independent biological replicates; N=3 per group; * denotes P ≤ 0.05 compared to respective control after unpaired t-test.*

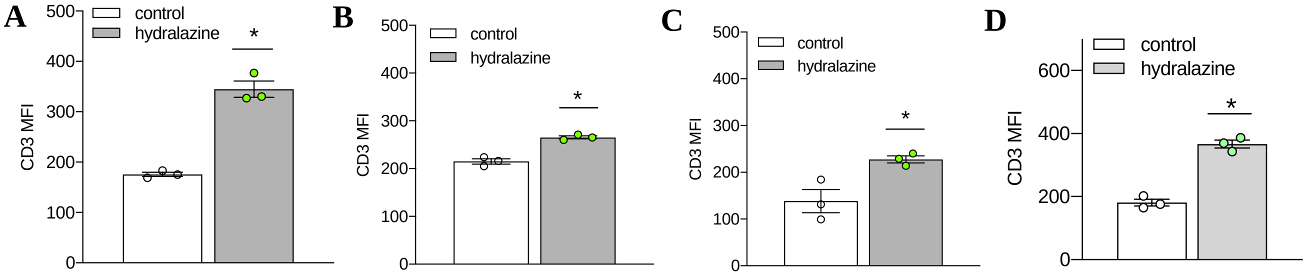

E

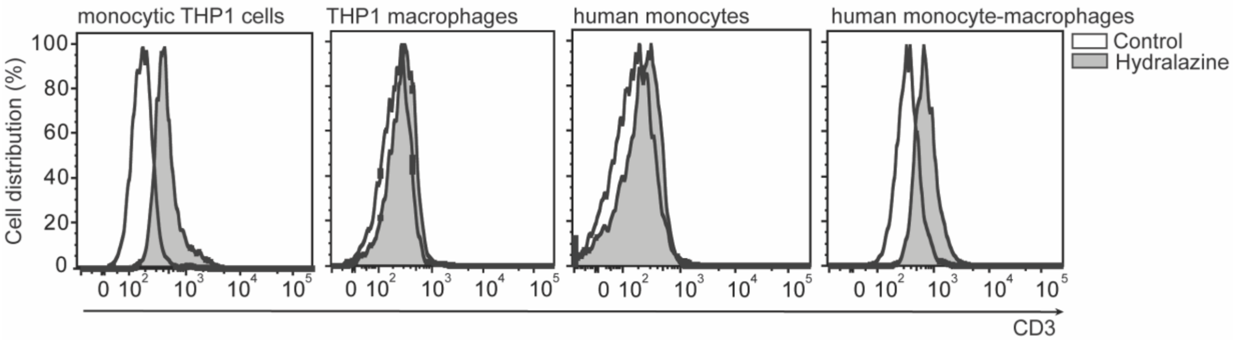

SUPPLEMENTARY FIGURE 5: Simvastatin and Hydralazine treatment have no effect on Jurkat cell activation. Flow cytometry analysis of Jurkat cells showing percentage of CD69$^+$ cells following LPS, simvastatin or hydralazine treatment (hydralazine – green circle, simvastatin – pink circles, combination – orange circles). LPS –lipopolysaccharide. *Values expressed in mean ± SEM; N denotes number of independent biological replicates; N=4-7 per group; Significance tested with Kruskal-Wallis followed by Dunn's post hoc testing.*

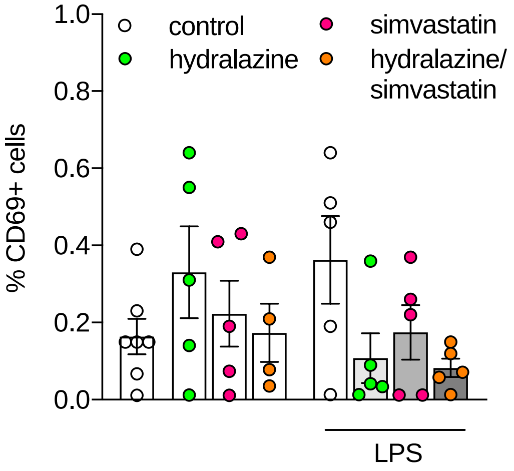

SUPPLEMENTARY TABLES

SUPPLEMENTARY TABLE 1: Comparison of memory function in young and aged WT and ApoE$^{-/-}$ mice. Behavioral parameters of WT and ApoE$^{-/-}$ mice obtained in novel object recognition tasks with 5-min or 24-hrs delay interval. RI as the main index of retention were calculated by the time spent investigating the novel object relative to the total object investigation [RI = $T_{Novel}/(T_{Novel} + T_{Familiar})$]. An RI=0.5 indicates that the animal has no preference for neither familiar nor novel object. ApoE – apolipoprotein E, RI – recognition index, WT –

wild-type. *Values expressed as mean+/- SEM; N denotes number of independent biological replicates; N=10 per group.* Mice with total object exploration times (T_{Novel} + $T_{Familiar}$) below 20s were excluded from the analysis. *Statistical differences were assessed with non-parametric Mann Whitney tests for single unpaired comparisons.*

	recognition index	P value	recognition index	P value
	5 min delay	compared to young control	24 hrs delay	compared to young control
WT young (4 months)	0.71 ± 0.05		0.57 ± 0.05	
WT aged (12 months)	0.74 ± 0.06	0.6403	0.58 ± 0.05	0.7974
ApoE$^{-/-}$ young (4 months)	0.76 ± 0.02		0.63 ± 0.02	
ApoE$^{-/-}$ aged (12 months)	0.73 ± 0.03	0.4584	0.36 ± 0.04	0.0009

SUPPLEMENTARY TABLE 2: Whole brain mRNA expression of inflammatory markers in 12-months old ApoE$^{-/-}$ versus 12-months old WT mice. ApoE – apolipoprotein E, CXCL – chemokine (C-X-C motif) ligand, GFAP – Glial fibrillary acidic protein, IL – interleukin, MCP-1 – monocyte chemoattractant protein 1, WT – wild-type. *Values are expressed as fold change compared to aged WT mice. N=5 per group; N denotes number of independent biological replicates.*

gene	Fold change (ApoE$^{-/-}$ vs. WT)
MCP-1	5.673
CXCL2	4.924
GFAP	1.969
CD68	2.927
IL6	5.507

SUPPLEMENTARY TABLE 3: Effect of Simvastatin and Hydralazine treatment on NeuN and BDNF expression in different brain regions of ApoE$^{-/-}$ mice. Quantification of immune stained NeuN+ and BDNF+ cells in hippocampus regions (CA3, CA1 region and DG) and cortex from all experimental groups. ApoE – apolipoprotein E, BDNF – brain-derived neurotrophic factor, CA – cornu ammonis, DG – dentate gyrus *Values are expressed as mean +/- SEM; N=3 for young and aged + combination treatment; N=4 for aged control and simvastatin treatment; N=5 for hydralazine treatment. N denotes number of independent biological replicates. * denotes P ≤ 0.05 relative to young control, & denotes P ≤ 0.05 relative to aged group after one-way ANOVA followed by Tukey's post hoc testing.*

	$ApoE^{-/-}$ young (4 months)	$ApoE^{-/-}$ aged (14 months)	$ApoE^{-/-}$ aged + hydralazine	$ApoE^{-/-}$ aged + simvastatin	$ApoE^{-/-}$ aged + hydralazine/simvastatin
hippocampus					
NeuN	1078.2 ± 59.8$^{\&}$	558.5 ± 55.8*	723.3 ± 69.8	797.5 ± 35.8*$^{\&}$	807.3 ± 50.1*$^{\&}$
BDNF	1484.0 ± 96.5$^{\&}$	438.5 ± 53.5*	675.0 ± 73.9	935.6 ± 37.7*$^{\&}$	751.0 ± 67.3
NeuN/BDNF ratio	1.377 ± 0.054$^{\&}$	0.781 ± 0.027*	0.953 ± 0.111*	1.297 ± 0.125$^{\&}$	0.971 ± 0.041*
cortex					
NeuN	768.0 ± 55.5	698.8 ± 55.9	940.8 ± 81.5	782.0 ± 39.3	939.0 ±50.2
BDNF	1650.0 ± 141.4	594.8 ± 68.0*	1266.0 ± 105.2$^{\&}$	1139.0 ± 171.9$^{\&}$	1151.0 ± 182.8$^{\&}$
NeuN/BDNF ratio	2.142 ± 0.032	0.845 ± 0.035*	1.364 ± 0.121	1.481 ± 0.265	1.213 ± 0.125

SUPPLEMENTARY TABLE 4: Effect of hydralazine and simvastatin treatment on monocyte and macrophage activation. Flow cytometry analysis (percentages) of THP-1 cells, primary monocytes and monocyte-derived macrophages treated with hydralazine or simvastatin. *For each experimental group N=3. N denotes number of independent biological replicates. * denotes P ≤ 0.05 compared to control and & denotes P ≤ 0.05 compared to hydralazine group after one-way ANOVA followed by Tukey's post hoc testing.*

	treatment	% CD3+ cells	% CD69+ cells
THP-1 monocytes	control	1.08 ± 0.04	4.60 ± 0.40
	10uM hydralazine	1.88 ± 0.09*	6.90 ± 0.66*
	1uM simvastatin	1.16 ± 0.07$^{\&}$	3.33 ± 0.37$^{\&}$
THP-1 macrophages	control	1.03 ± 0.10	2.88 ± 0.25
	10uM hydralazine	2.11 ± 0.14*	4.53 ± 0.18*
	1uM simvastatin	1.27 ± 0.18$^{\&}$	2.40 ± 0.17$^{\&}$
Human primary monocytes	control	2.56 ± 0.23	1.70 ± 0.13
	10uM hydralazine	4.50 ± 0.72*	6.48 ± 0.38*
	1uM simvastatin	2.80 ± 0.35	2.30 ± 0.36
Human primary monocyte-derived macrophages	control	6.83 ± 0.33	8.07 ± 0.43
	10uM hydralazine	16.00 ± 1.16*	9.20 ± 0.51
	1uM simvastatin	8.73 ± 0.62	6.33 ± 0.32*

***SUPPLEMENTARY TABLE 5:* List of antibodies for flow cytometry.**

Target	Host/species	Label/dye	clone	dilution	distributor
CD11b	Rat anti-mouse	PE-Texas Red	M1/70.15	1:100	Life Technology
CD3	Rat anti-mouse	eFluor450	17A2	1:100	eBioscience
CD45	Rat anti-mouse	AF700	30-F11	1:100	eBioscience
CD45R	Rat anti-mouse	AF488	RA3-6B2	1:100	BioTechne
Ly6C	Rat anti-mouse	PE-Cy7	HK1.4	1:100	eBioscience
Ly6G	Rat anti-mouse	APC	RB6-8C5	1:100	eBioscience
CD14	Anti-human	eFluor450	61D3	1:100	Life Technology
CD16	Anti-human	FITC	CB16	1:100	Life Technology
CD3	Anti-human	FITC	BW264/56	1:100	Miltenyi Biotec
CD69	Anti-human	APC	298614	1:100	Life Technology
Live/dead		Aqua-405		1:250	Life Technology

***SUPPLEMENTARY TABLE 6:* List of antibodies used for immunofluorescence.**

Antibody target	host	Supplier (catalogue #)	Antibody dilution
BDNF	rabbit	Abcam (ab6201)	1:250
NeuN	mouse	Abcam (ab104224)	1:250
Iba-1	rabbit	WAKO (019-19741)	1:1000
CD68 (ED-1)	rat	Invitrogen (MA5-16654)	1:500

***SUPPLEMENTARY TABLE 7:* List of antibodies used for Western blotting.**

Antibody target	host	Supplier (catalogue #)	Antibody dilution
CD3	rabbit	Abcam (ab5690)	1:1000
Beta-tubulin	mouse	Sigma Aldrich (T4026)	1:3000
SNAP-25	rabbit	Abcam (ab109105)	1:5000
PSD-95	rabbit	Abcam (ab76115)	1:2000

SUPPLEMENTARY TABLE 8: **List of mouse and human primers used for qPCR.**

Target	Primer sequence (mouse)
L14	F: 5'-GGCTTTAGTGGATGGACCCT-3’<br>R: 5'-ATTGATATCCGCCTTCTCCC-3'
TBP	F: 5’-GAAGCTGCGGTACAATTCCAG-3’<br>R: 5’-CCCCTTGTACCCTTCACCAAT-3’
IL12	F: 5'-CATCTGGTTCAGTGCTTTGATCT-3’<br>R: 5'-ACCCGTGAGTTATTCCATGAGT-3'
MCP-1	F: 5'-CACGACATGCACGTTTGACT-3’<br>R: 5'-GGCGCAGATAAGGCTTCACA-3'
TNF-a	F: 5'-GTTTGCAGTCTCTCAAGCTTTT-3’<br>R: 5'-CCGATTTGAGCAATCGTTT-3'
IL6	F: 5'-AGGAGTGATACCAGCTTTAGTCC-3’<br>R: 5'-CCGAGCAGGTCAGAACAAAGG-3'
iNOS	F: 5'-GGC AGC CTG TGA GAC CTT TG-3'<br>R: 5'-GCA TTG GAA GTG AAG CGT TTC-3'
CXCl2	F: 5'-GTCCCAGACATCAGGGAGTAA-3’<br>R: 5'-TCGGATACTTCAGCGTCAGGA-3'
IL23	F: 5'-GACCCACAAGGACTCAAGGAC-3’<br>R: 5'-ATGGGGCTATCAGGGAGTAGAG-3'
Arg-1	F: 5'-TTGCGAGACGTAGACCCTGG-3’<br>R: 5'-CAAAGCTCAGGTGAATCGGC-3'
CD68	F: 5'-TGTCTGATCTTGCTAGGACCG-3’<br>R: 5'-GAGAGTAACGGCCTTTTTGTGA-3'
CD86	F: 5'-AGAACTTACGGAAGCACCCAC-3’<br>R: 5'-CTGCCAAAATACTACCAGCTCAC-3'
PSD-95	F: 5'-ACCGCTACCAAGATGAAGACAC-3’<br>R: 5'-CTCCTCATACTCCATCTCCCC-3'
BDNF	F: 5'-TGCAGGGGCATAGACAAAAGG-3’<br>R: 5'-CTTATGAATCGCCAGCCAATTCTC-3'
**Target**	**Primer sequence (human)**
GPI	F: 5'-AGG CTG CTG CCA CAT AAG GT-3’<br>R: 5'-CCA AGG CTC CAA GCA TGA AT-3'

IFN-γ	F: 5'-ACTGACTTGAATGTCCAACGCA-3’<br>R: 5'-ATCTGACTCCTTTTTCGCTTCC-3'
IL-6	F: 5'-TGC GTC CGT AGT TTC CTT CT-3’<br>R: 5'-GCC TCA GAC ATC TCC AGT CC-3'

Gating strategy

A. Brain

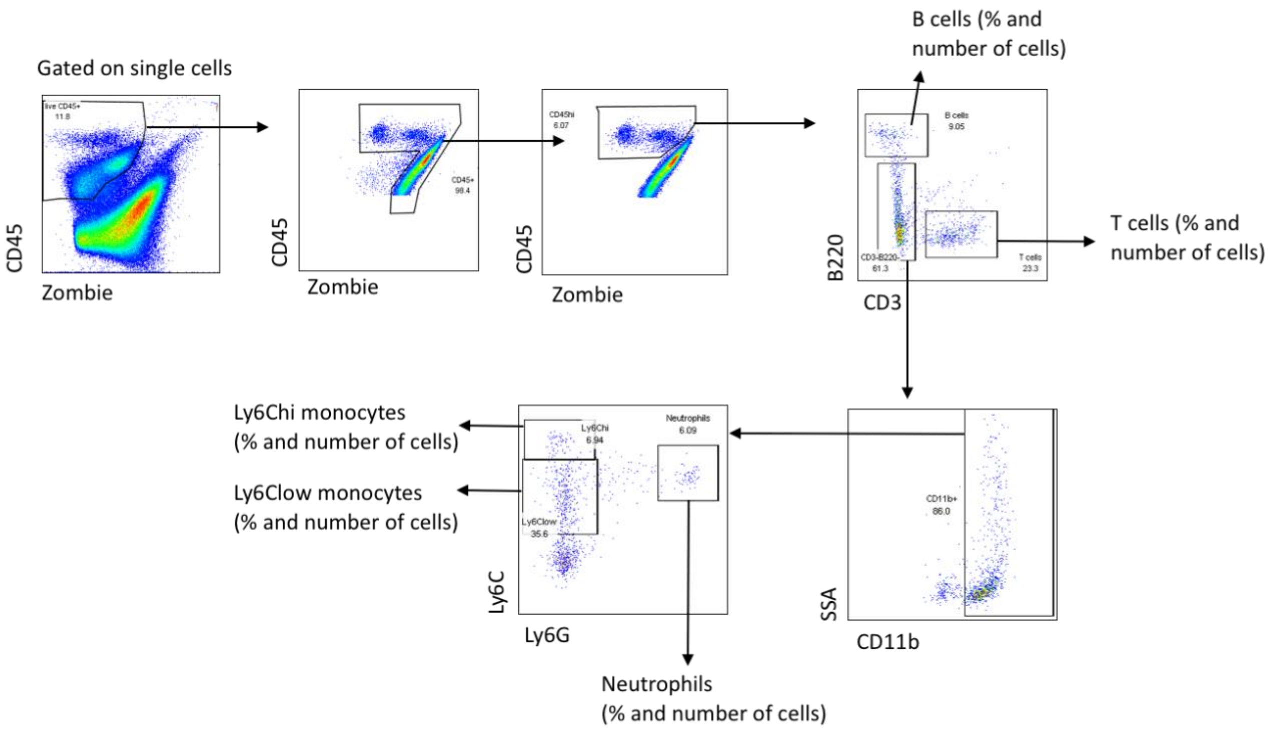

B. Blood

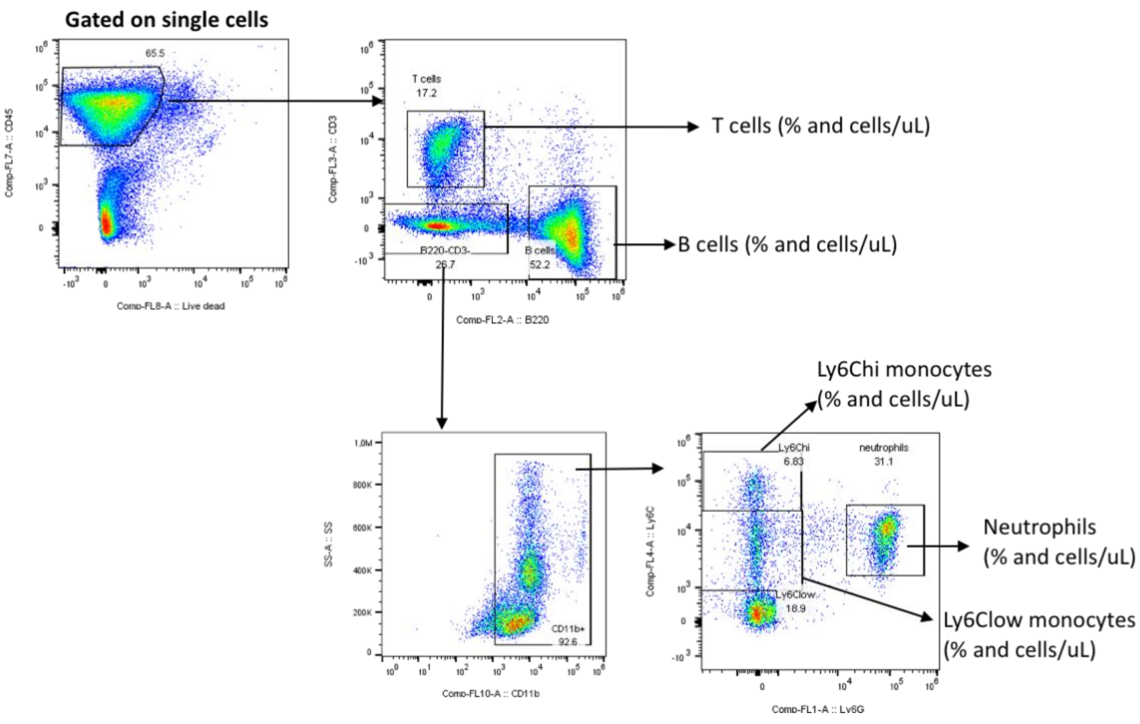

Western blot images (representative bands used for insets in main figures highlighted with red borders)

*Note: Full blots without cropping are given below. For SNAP-25 and PSD-95, molecular weight markers do not appear on the membranes because special stain-free markers were used in our system. To verify the protein sizes, a bright field image is obtained from the same membrane to overlay for analysis. The merged images are given below the membrane-only images. For CD3, the molecular weight marker is showing on the right corner of the membrane. The corresponding tubulin signal is too strong (1 sec exposure time) to visualize any protein standard band.

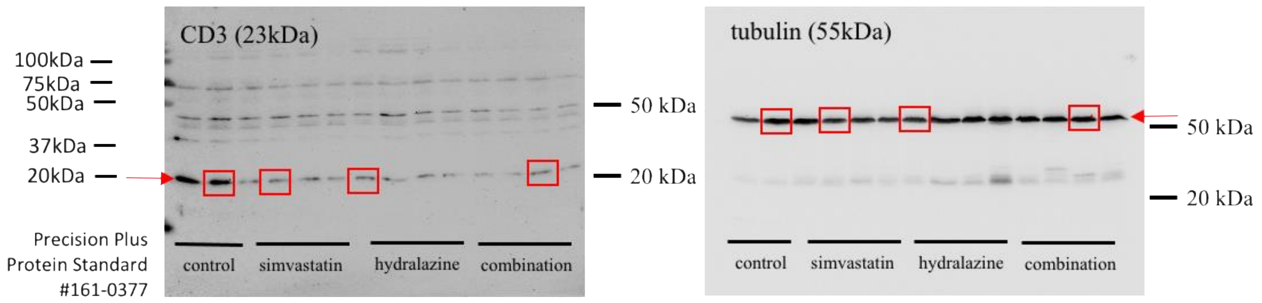

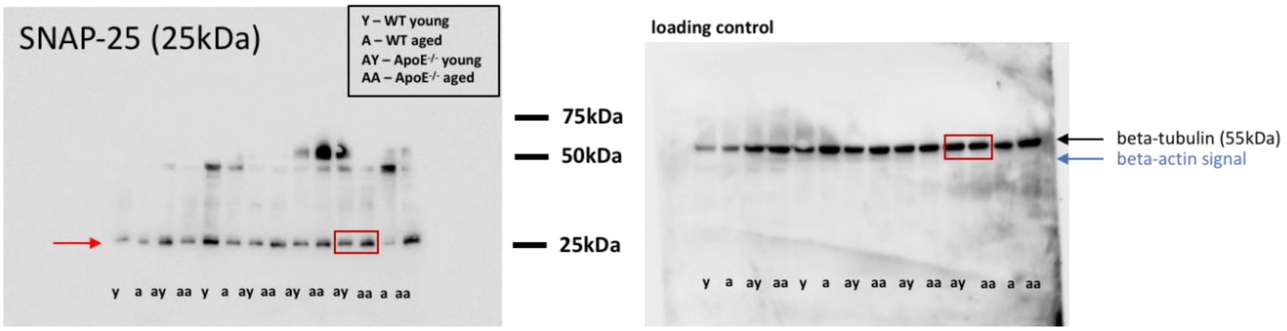

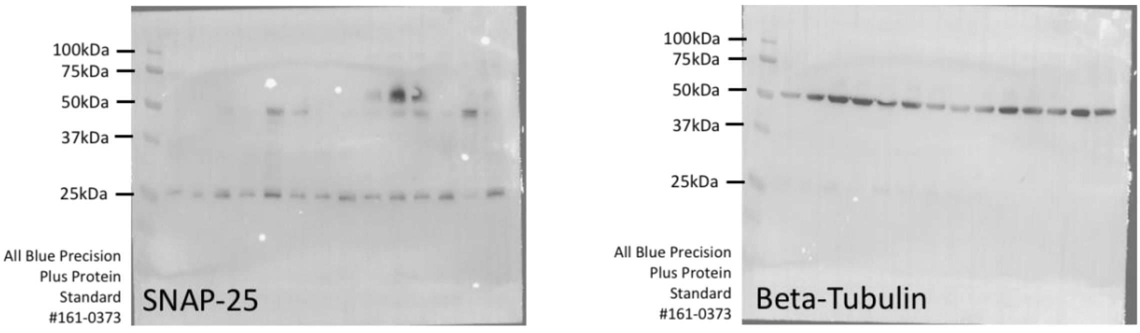

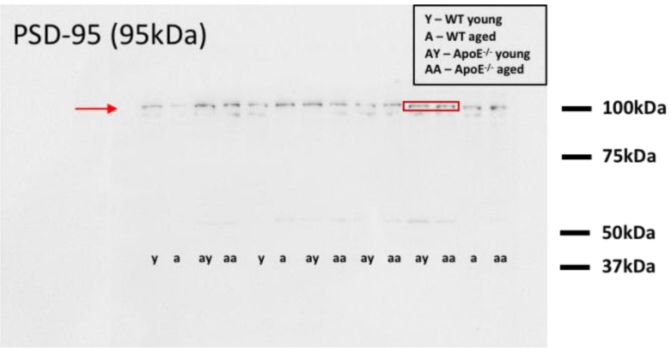
PSD-95 (95kDa)
Y – WT young
A – WT aged
AY – $ApoE^{-/-}$ young
AA – $ApoE^{-/-}$ aged
100kDa
75kDa
50kDa
37kDa
y a ay aa y a ay aa ay aa ay aa a aa

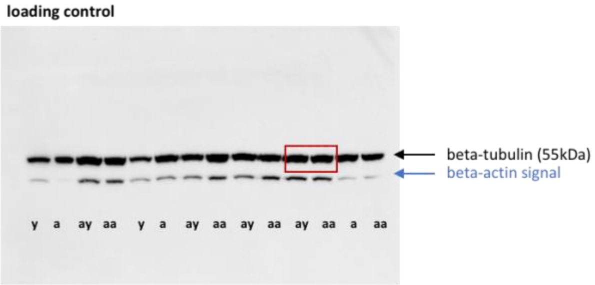
loading control
beta-tubulin (55kDa)
beta-actin signal
y a ay aa y a ay aa ay aa ay aa a aa

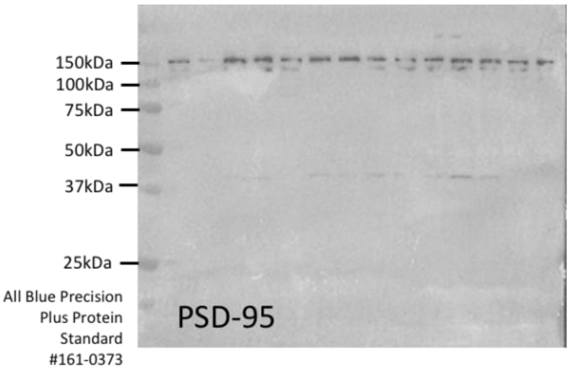
150kDa
100kDa
75kDa
50kDa
37kDa
25kDa
All Blue Precision Plus Protein Standard #161-0373
PSD-95

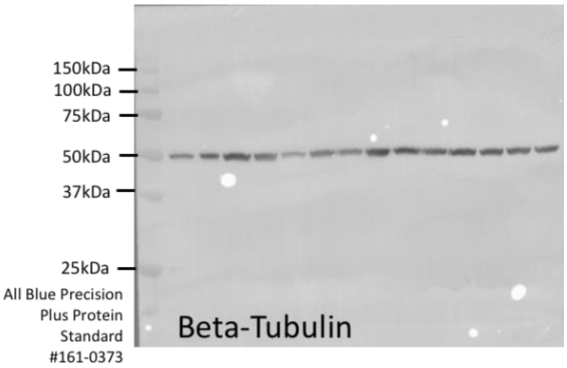
150kDa
100kDa
75kDa
50kDa
37kDa
25kDa
All Blue Precision Plus Protein Standard #161-0373
Beta-Tubulin
