# Supplementary material for: Simvastatin therapy attenuates memory deficits that associate with brain monocyte infiltration in chronic hypercholesterolemia
Source: NPJ Aging Mech Dis. 2021 Aug 4;7:19. doi: 10.1038/s41514-021-00071-w (PMC8338939; doi:10.1038/s41514-021-00071-w)
Supplement: Supplementary file 2 — Reporting Summary [file 41514_2021_71_MOESM2_ESM.pdf]

nature research

Corresponding author(s): Anja Meissner

Last updated by author(s): Feb 13, 2021

Reporting Summary

Nature Research wishes to improve the reproducibility of the work that we publish. This form provides structure for consistency and transparency in reporting. For further information on Nature Research policies, see our Editorial Policies and the Editorial Policy Checklist.

Statistics

For all statistical analyses, confirm that the following items are present in the figure legend, table legend, main text, or Methods section.

n/a	Confirmed	
☐	☒	The exact sample size (*n*) for each experimental group/condition, given as a discrete number and unit of measurement
☐	☒	A statement on whether measurements were taken from distinct samples or whether the same sample was measured repeatedly
☐	☒	The statistical test(s) used AND whether they are one- or two-sided<br>*Only common tests should be described solely by name; describe more complex techniques in the Methods section.*
☒	☐	A description of all covariates tested
☐	☒	A description of any assumptions or corrections, such as tests of normality and adjustment for multiple comparisons
☐	☒	A full description of the statistical parameters including central tendency (e.g. means) or other basic estimates (e.g. regression coefficient) AND variation (e.g. standard deviation) or associated estimates of uncertainty (e.g. confidence intervals)
☒	☐	For null hypothesis testing, the test statistic (e.g. *F*, *t*, *r*) with confidence intervals, effect sizes, degrees of freedom and *P* value noted<br>*Give P values as exact values whenever suitable.*
☒	☐	For Bayesian analysis, information on the choice of priors and Markov chain Monte Carlo settings
☒	☐	For hierarchical and complex designs, identification of the appropriate level for tests and full reporting of outcomes
☐	☒	Estimates of effect sizes (e.g. Cohen's *d*, Pearson's *r*), indicating how they were calculated

Our web collection on statistics for biologists contains articles on many of the points above.

Software and code

Policy information about availability of computer code

Data collection	*Provide a description of all commercial, open source and custom code used to collect the data in this study, specifying the version used OR state that no software was used.*
Data analysis	All statistical analyses were carried out using GraphPad software (Version 8.4.2).

For manuscripts utilizing custom algorithms or software that are central to the research but not yet described in published literature, software must be made available to editors and reviewers. We strongly encourage code deposition in a community repository (e.g. GitHub). See the Nature Research guidelines for submitting code & software for further information.

Data

Policy information about availability of data

All manuscripts must include a data availability statement. This statement should provide the following information, where applicable:

- Accession codes, unique identifiers, or web links for publicly available datasets
- A list of figures that have associated raw data
- A description of any restrictions on data availability

The data underlying this article are available in the article and in its online supplementary material.

Field-specific reporting

Life sciences study design

All studies must disclose on these points even when the disclosure is negative.

Sample size	Experimental groups were designed in a way to minimize stress for the animals and to guarantee maximal information using the lowest group size possible using a power calculation with Type I error α= 0.05 and Power of 1-β > 0.8 (80%) based on previous studies
Data exclusions	For brain FACS experiments, samples with high myelin contamination were excluded from analysis. This is indicated in the respective figure legends.
Replication	In vitro studies were repeated a minimum of five times with each experimental condition run in triplicates. N denotes number of independent biological replicates.
Randomization	Mice were randomly assigned to the following experimental groups using the computer software Research Randomizer (http:// www.randomizer.org/)
Blinding	To ensure blinding experiments were performed after the animals and samples had received codes that did not reveal the identity of the treatment.

Reporting for specific materials, systems and methods

We require information from authors about some types of materials, experimental systems and methods used in many studies. Here, indicate whether each material, system or method listed is relevant to your study. If you are not sure if a list item applies to your research, read the appropriate section before selecting a response.

Materials & experimental systems

n/a	Involved in the study	
☐	☒	Antibodies
☐	☒	Eukaryotic cell lines
☒	☐	Palaeontology and archaeology
☐	☒	Animals and other organisms
☒	☐	Human research participants
☒	☐	Clinical data
☒	☐	Dual use research of concern

Methods

n/a	Involved in the study	
☒	☐	ChIP-seq
☐	☒	Flow cytometry
☒	☐	MRI-based neuroimaging

Antibodies

Antibodies used

Commercially available primary antibodies against CD3 (Bio-techne, MAB4841), PSD-95 (Abcam, ab18258), SNAP-25 (Abcam, ab5666), NeuN (Abcam, ab104225) and BDNF (Abcam, ab203573), CD68 (Invitrogen, MA5-16654) and Iba-1 (Wako, 4987481428584) were used for immunofluorescence. Secondary antibodies Alexa Fluor 488 donkey anti-mouse, anti-rabbit, goat anti-rabbit or goat anti-rat Fluor 594 (Nordic Biosite, Sweden) were used for visualization.

Validation

CD68 (Invitrogen, MA5-16654): Neural precursor cells form integrated brain-like tissue when implanted into rat cerebrospinal fluid. "MA5-16654 was used in Immunohistochemistry to report that early stage neural precursor cells recapitulate their seminal properties and develop into large brain-like tissue when implanted into the rat brain ventricle." Pothayee N,Maric D,Sharer K,Tao-Cheng JH,Calac A,Bouraoud N,Pickel J,Dodd S,Koretsky A. 2020

NeuN (Abcam, ab104225): Matta SM et al. An altered glial phenotype in the NL3R451C mouse model of autism. Sci Rep 10:14492 (2020).

BDNF (Abcam, ab203573): Vidal-Martinez G et al. FTY720 Improves Behavior, Increases Brain Derived Neurotrophic Factor Levels and Reduces a-Synuclein Pathology in Parkinsonian GM2+/- Mice. Neuroscience 411:1-10 (2019).

PSD-95 (Abcam, ab18258): Zhang J et al. Metformin treatment improves the spatial memory of aged mice in an APOE genotype-dependent manner. FASEB J 33:7748-7757 (2019).

Iba-1 (Wako, 4987481428584): Fantin, A., et al.: Blood, 116, 5, 829 (2010); Ising, C., et al.: Nature, 575, 669(2019); Jacob, F., et al.: Cell. 180, 188(2020).

CD3 (Bio-techne, MAB4841): https://www.bio-techne.com/p/antibodies/mouse-cd3-antibody-17a2_mab4841#scientific-data-anchor

SNAP-25 (Abcam, ab5666):

Radford H   et al. PERK inhibition prevents tau-mediated neurodegeneration in a mouse model of frontotemporal dementia. Acta Neuropathol 130:633-42 (2015).

Eukaryotic cell lines

Policy information about cell lines

Cell line source(s)	Human monocytic THP-1 cells (ATCC #TIB-202)
Authentication	STR profiling:<br>Amelogenin: X,Y<br>CSF1PO: 11,13<br>D13S317: 13<br>D16S539: 11,12<br>D5S818: 11,12<br>D7S820: 10<br>TH01: 8,9.3<br>TPOX: 8,11<br>vWA: 16
Mycoplasma contamination	negative mycoplasma tests for both cell lines
Commonly misidentified lines (See ICLAC register)	not applicable

Animals and other organisms

Policy information about studies involving animals; ARRIVE guidelines recommended for reporting animal research

Laboratory animals	Male wild-type (WT) C57Bl/6J mice and Apolipoprotein E knockout (ApoE-/-) mice (B6.129P2-Apoe (tm1Unc)/J) were obtained from Jackson Laboratories and bred in a conventional animal facility under standard conditions with a 12h:12h light-dark cycle, and access to food (standard rodent diet) and water ad libitum.
Wild animals	N/A
Field-collected samples	N/A
Ethics oversight	institutional ethical committee of Lund University (Dnr. 5.8.18/12637/2017)

Note that full information on the approval of the study protocol must also be provided in the manuscript.

Flow Cytometry

Plots

Confirm that:

- [x] The axis labels state the marker and fluorochrome used (e.g. CD4-FITC).
- [x] The axis scales are clearly visible. Include numbers along axes only for bottom left plot of group (a 'group' is an analysis of identical markers).
- [x] All plots are contour plots with outliers or pseudocolor plots.
- [x] A numerical value for number of cells or percentage (with statistics) is provided.

Methodology

Sample preparation	Whole blood from mice was collected in EDTA-coated tubes and red blood cells were lysed before samples were incubated in Fc block solution followed by primary antibodies. After centrifugation, the supernatant was decanted, washed, and pellets were re-suspended in FACS buffer. Brain tissue was enzymatically digested and homogenized. After density separation using Percoll (GE Healthcare), pellets were reconstituted in Fc block prior to staining with antibodies (see data supplement for detailed list of antibodies). Data acquisition was carried out in a BD LSR Fortessa cytometer using FacsDiva software Vision 8.0 (BD Biosciences). Data analysis was performed with FlowJo software (version 10, TreeStar Inc., USA). Cells were plotted on forward versus side scatter and single cells were gated on FSC-A versus FSC-H linearity.
Instrument	BD LSR Fortessa cytometer
Software	FacsDiva software Vision 8.0 (BD Biosciences). Data analysis was performed with FlowJo software (version 10, TreeStar Inc., USA)
Cell population abundance	N/A
Gating strategy	Cells were plotted on forward versus side scatter and single cells were gated on FSC-A versus FSC-H linearity. Positive and

negative populations were set based on signals obtained from antibody-specific signals using compensation beads.

☒ Tick this box to confirm that a figure exemplifying the gating strategy is provided in the Supplementary Information.
